# Quality assessment of butter cookies applying multispectral imaging

**DOI:** 10.1002/fsn3.46

**Published:** 2013-06-12

**Authors:** Mette S Andresen, Bjørn S Dissing, Hanne Løje

**Affiliations:** National Food Institute, Technical University of DenmarkSøltofts Plads 227, DK-2800, Kongens Lyngby, Denmark

**Keywords:** Baking, browning, cookie, food technology, food quality, multispectral imaging, water content

## Abstract

A method for characterization of butter cookie quality by assessing the surface browning and water content using multispectral images is presented. Based on evaluations of the browning of butter cookies, cookies were manually divided into groups. From this categorization, reference values were calculated for a statistical prediction model correlating multispectral images with a browning score. The browning score is calculated as a function of oven temperature and baking time. It is presented as a quadratic response surface. The investigated process window was the intervals 4–16 min and 160–200°C in a forced convection electrically heated oven. In addition to the browning score, a model for predicting the average water content based on the same images is presented. This shows how multispectral images of butter cookies may be used for the assessment of different quality parameters. Statistical analysis showed that the most significant wavelengths for browning predictions were in the interval 400–700 nm and the wavelengths significant for water prediction were primarily located in the near-infrared spectrum. The water prediction model was found to correctly estimate the average water content with an absolute error of 0.22%. From the images it was also possible to follow the browning and drying propagation from the cookie edge toward the center.

## Introduction

In cookie production, visual quality is of very high importance. To ensure that the quality is constantly in accordance with the specifications, it is inspected regularly throughout the production day, so that the process can be adjusted immediately if needed. Traditionally, such control and quality checking is done by human expert ope-rators. Even though it can be advantageous to have expert human operators placed directly at the production line, there are also some disadvantages. Besides being labor intensive, manual inspectors might evaluate the products slightly different, they are also by nature subjective, and their evaluations may be inconsistent so that they vary over time, even for the same expert (Little 1973). Therefore, alternative fast and objective control systems are desirable and it is not surprising that the development of fast nondestructive methods for food product quality monitoring is a rapidly evolving field. A few examples can be found in Jha et al. (2011), Telis-Romero et al. (2011), Liao et al. (2012), Oto et al. (2012), discussing such methods as UV spectra, electrical properties, ultrasound, and near-infrared (NIR) spectroscopy for online product quality assessment.

The surface color is one of the first impressions consumers get of a bakery product (Clydesdale 1991), giving the surface browning of cookies high importance as a quality parameter. The surface color of bakery products depends both on the physicochemical characteristics of the raw dough, such as water content, pH, reducing sugars, and amino acid content, and on the operating conditions during processing, mainly the final baking (Purlis 2011). During baking, the temperature, time, air velocity, and relative humidity can influence the browning of cookies.

Equally important, but harder to evaluate by online methods, is the water content of the cookie. If the water content is too high the shelf life of the product will be impaired (Manley and Clark 2011). During baking, water is transported through and away from the product. Evaporation takes place at the surfaces, and water migrates through the product toward the lower concentration at the surface. A low water activity is a prerequisite for browning. While the water activity is not identical to the water content, as water activity is a measure of free water and water content is the total amount of water in the product, the two are closely related (Cauvain and Young 2000). For the purpose of this study, the water content has been used. To some extent, the water content might be deducted from the degree of browning; however, the opposite is not always the case, as a low water content can be obtained without browning at low surface temperatures (approximately below 115°C) (Broyart et al. 1998).

The purpose of this work was to investigate the use of multispectral image analysis to assess multiple quality aspects of bakery products from the same image. The investigated product is a butter cookie, and the investigated quality parameters are the surface browning and the water content after cooling. The developed browning score is based on sensory evaluation, distributing cookies in different browning categories. Combining surface browning with sensory evaluations, forming a single browning score, which is modeled as a function of the baking time and oven temperature has, to the knowledge of the authors, not been presented in previous academic papers. The ability to quantify the water content from the same multispectral images is furthermore investigated, showing that it is possible to assess different quality parameters from the same multispectral image and thereby saving time compared to the process of obtaining the information through visual observations or laboratory work.

In this work, the investigation is focused on the influence of baking time and oven temperature on browning and moisture content. A similar study of biscuits was presented in Carstensen (2012). In this work, a browning index was considered, along with the average water content. Additionally, a glazing index was obtained. The ability to predict the water content was found to be 0.14% root mean squared error of prediction (RMSEP) (see eq. 2). This study investigated industrially produced biscuits, limiting the investigated water content range to 1–3%. The browning index was built using endpoints, these being conforming and nonconforming areas of biscuits. A Fischer's discriminant analysis (FDA) was then used to map biscuits into a scale between these endpoints.

In the present work, a range of processing conditions (varying the time and temperature) were investigated. Compared to the study investigating biscuits produced in an industrial setting without variations, the present study investigates a broader window of applicability for the method. In the present work, the browning of the butter cookies ranged from underbaked to overbaked and the average water content ranged from 1.1% to 9.4%.

## Materials and Methods

The present work is focused on two cookie data sets. The first set, Set 1, was prepared and used for sensory evaluation and model development with regard to the browning score. The second set, Set 2, was used to validate the browning model, build the browning response surface model, and to build and validate the water content model. The degree of browning was estimated on the second baking set, based on the model developed from Set 1. The water content was measured and estimated for Set 2. For water content evaluation, Set 2 was split into two groups, a modeling and an evaluation group. In the following, the baking procedure, the evaluation of browning, the method for water content measurements, the multispectral image capturing equipment, and the data processing methods are described.

### Baking procedure

The examined butter cookies were prepared following the recipe in Table 1, provided by Haas-Meincke A/S^1^ as an example of a typical industrial cookie product. The ingredients were mixed using a Varimixer Teddy (5L) mixer, (A/S Wodschow & Co., Broendby, Denmark) and rolled using a Rollmatic manual sheeter (Rollmatic Srl, Schio, Italy). The dough was rolled to a height of 6 mm (±0.5 mm) and the cookies were cut to a diameter of 45 mm. The same cutter was used for all cookies. Between shaping and baking, the cookies were stored cool (5°C) and covered by plastic film to avoid dehydration of the surface. The cookies were baked in a Rational Combi CCC convection oven (Rational AG, Landsberg a. Lech, Germany).

**Table 1:** Composition and mixing procedure for 1.7 kg cookie dough

Ingredients	Mass (g)
Icing sugar	333
Margarine	500
Vanilla extract	3.3
Mix until	Even texture
Whole egg	33.3
Skimmed milk powder	12.5
Salt	3.3
Sodium bicarbonate	0.4
Water	8.3
Mix until	Even texture
Corn starch	167
Wheat cake flour	667
Mix until an even	Textured dough is obtained
Finish by gathering	The dough by hand
Total weight	1728

The experimental part of the work was centered around the construction of two independent data sets. The first data set (Set 1) was created both as a training set for the multispectral imaging device and to be used in a sensory evaluation of the appearance of the cookies. Three cookies were used for imaging for each baking time. These images were used to create a browning score which indicates the stage of browning of a specific cookie. The cookies in this set were baked at a fixed temperature (180°C) varying the baking time to produce a range of browning.

The second data set (Set 2) was used to build a response surface describing how the time and temperature affects the browning score. The cookies for this set were baked at different oven temperatures and with varying baking times. Triplicates were made of all imaged cookies.

All cookies in Set 1 were baked at 180°C varying the baking time to obtain varied degrees of browning. The baking times were: 4, 6, 7, 8, 9, 10, 12, 14, 16, and 20 min. Previous experiments by the authors had shown that the degree of browning moved from covering only the edge of the cookie to the whole surface at baking times between 6 and 9 min. Therefore, 1 min intervals were applied in this range.

The cookies in Set 1 were baked at different air temperatures (150, 160, 170, 180, and 200°C) and at different baking times (4, 6, 8, 10, 12, 14, and 16 min). These parameters were chosen to cover a wide range of surface browning.

Other parameters such as dough composition, product dimension, cooling time, and oven settings (air humidity and air speed) were kept constant for all baking experiments.

### Evaluation of browning

In the current work, the goal was to link a sensory evaluation to a calculated browning score for the butter cookies. A panel consisting of six untrained persons was used to assess the degree of browning. In a production planning, a larger or trained panel might be used for a full-scale sensory analysis. In the present work, the objective of the analysis was to demonstrate a principle. Therefore, the smaller panel is well suited.

The panelists were in their mid-20s to mid-30s of Danish decent with a good knowledge of food production processes and food quality. The cookies from Set 1 were presented to the panel without information on the objectives of the study or the variations in baking times. Each panelist was asked to group the cookies into three categories, independently from the other members in the panel, using a form. The only criterion for separation was the appearance of the cookies with regard to browning. The evaluation was done in a daylight setting. The panelists were asked to divide the cookies into the following three groups: *underbaked*, *adequately baked*, and *overbaked*. Each cookie could only be assigned to one category.

### Water content

The water content was determined for Set 2. The average water content was found in the cookies after baking and cooling. The weight of the cookies was recorded before and after the cookies were placed in a drying cabinet at 105°C for 48 h (or until no further changes were registered in the weight). The mass loss during drying is assumed to be equal to the total water content in the baked cookie before drying. The final weight is seen as the dry matter (DM) content of the cookie.

In this work the water content is reported as the average water content or percentage of water per cookie. The fraction water per total cookie weight (*X*) is calculated as seen in equation 1:


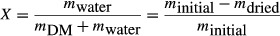
(1)

where *m* is the weight of a given component in the cookie – either DM, water, or the initial or dried weight of the cookie.

### VideometerLab

**Figure 1:**
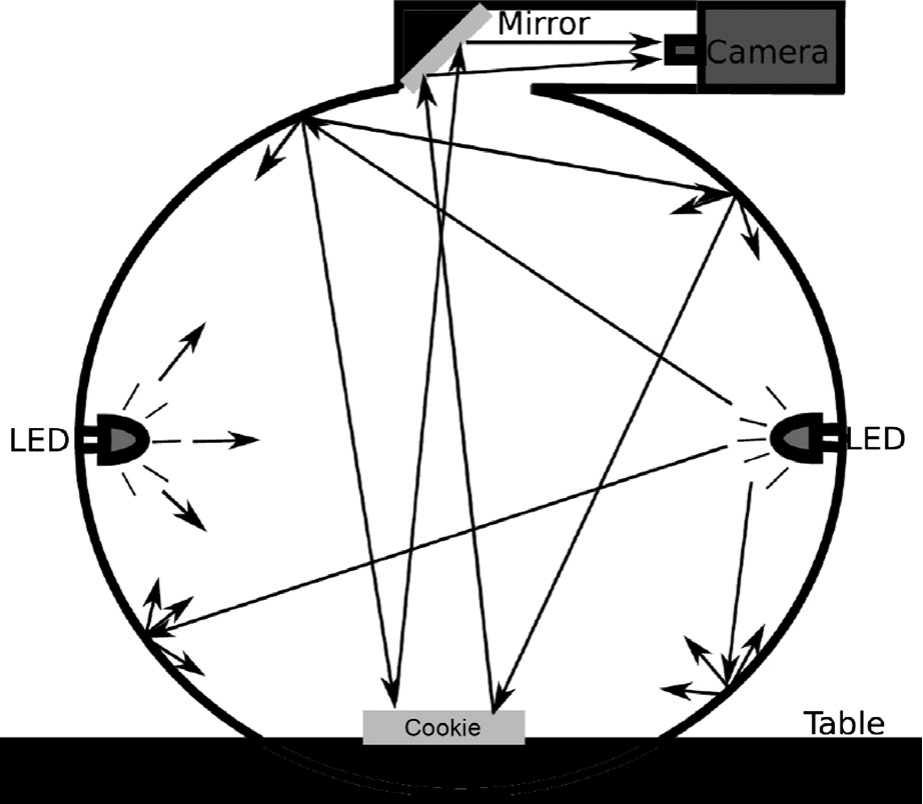
Schematic illustration of the Videometerlab setup showing the Ulbricht sphere, light-emitting diodes (LEDs), and camera positions (Dissing et al. 2011).

The imaging data was acquired using a VideometerLab (Carstensen et al. 2006). The VideometerLab captures multispectral images at 19 different wavelengths ranging from 385 to 970 nm. The system records the surface reflections with a standard monochrome charged coupled device chip, nested in a Point Grey Scorpion camera (Point Grey Research GmbH, Ludwigsburg, Germany). A schematic illustration of the whole setup is shown in Figure 1. The item of interest is placed inside a sphere with a matte white coating, an integrating or so called Ulbricht sphere. Inside the sphere light-emitting diodes (LED) are placed side by side in a pattern distributing the LEDs of each wavelength evenly across the whole perimeter to avoid shadows and specular reflections. During data acquisition, the diodes are strobing successively resulting in a 1280 × 960 image for each wavelength. Before data acquisition is commenced, the system is calibrated both radiometrically and geometrically followed by a light setup, to obtain the optimal dynamic range for all LEDs, as well as minimizing the distortions in the lens and thereby pixel correspondence across the spectral bands (Carstensen and Folm-Hansen 2000). The system is developed to guarantee the reproducibility of collected images, which means that it can be used in comparative studies of time series, or across a large variety of different samples.

In other recent studies, the VideometerLab setup was applied for quality studies of food products, as a fast method to quantify the amount of the red colored astaxanthin in salmonids (Dissing et al. 2011), to measure the degree of agglutination in continuously fried minced meat (Daugaard et al. 2010), and as a method to evaluate quality changes in stored wok fried vegetables (Clemmesen et al. 2012). According to the company Videometer A/S^2^ the VideometerLab is currently implemented in industrial quality control lines including color sorting of mink furs (Kopenhagen Fur, Copenhagen, Denmark) and monitoring shape and texture distribution of bulk granule samples (Ferring International Center S.A., Saint-Prex, Switzerland).

#### Calculation of browning score

Multispectral images were treated to first remove the background, leaving only pixels with cookie surface. This was done using simple adaptive thresholding techniques. The browning score based on multispectral images and sensory evaluations is created using FDA (Hastie et al. 2001). The images in Set 1 were divided into categories by the sensory panel. Based on this division, the images were transformed to multivariable data matrices containing only pixel values from the surface of the cookies. The within-group and between-group scattering matrices used in the FDA were estimated based on all pixel values across the cookie surfaces. Using the two scattering matrices, an optimal projection vector was found so that each group, when projected onto the vector, contracted so that the three groups were scattered as much as possible. In this way, the separation between the categories within the browning score space (which we call the space, in which the cookies are projected into) was maximized.

### Estimating water content from images

In addition to the browning index, it was investigated whether the available NIR spectrum may be used to estimate the water content in the cookies. NIR imaging has in other studies proved to be a good method for estimation of water content in food products, such as minced meat (Dissing et al. 2009), breaded chicken nuggets (Yavari et al. 2011), and water-related qualities, such as drip loss (Barbin et al. 2012). Multispectral imaging is capable of detecting water shifts in the NIR area of the electromagnetic spectrum, disregarding the surface color. The brown color on the surface of the products is known to have high absorbance properties in the UV and visual areas (Kim and Lee 2009), which are well separated from the water peaks at 970, 1440, and 1930 nm.

To investigate the relation between the measured water content and the recorded spectra, a prediction model was created based on the mean spectra of cookies in Set 2, and the corresponding measured average water content (as given in eq. 1). A partial least squares regression (PLSR) was used to build the prediction model based on 2/3 of the available data, and tested on the remaining third of the data set (Wold et al. 2001). Parameters were estimated from a fivefold cross-validation scheme using the larger group of the data set. The accuracy of the model is given as the RMSEP, calculated as shown in equation 2 (Esbensen et al. 2002):


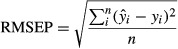
(2)

where 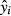 is the predicted average water content per cookie, *y* is the average measured water content, and *n* is the number of samples.

## Results and Discussion

The results for the investigations of browning and moisture content are presented separately in the following sections. The first presented results are concerned with the browning and the browning model, the subsequent sections deal with the moisture content, the prediction model, and the results.

### Multispectral images

**Figure 2:**
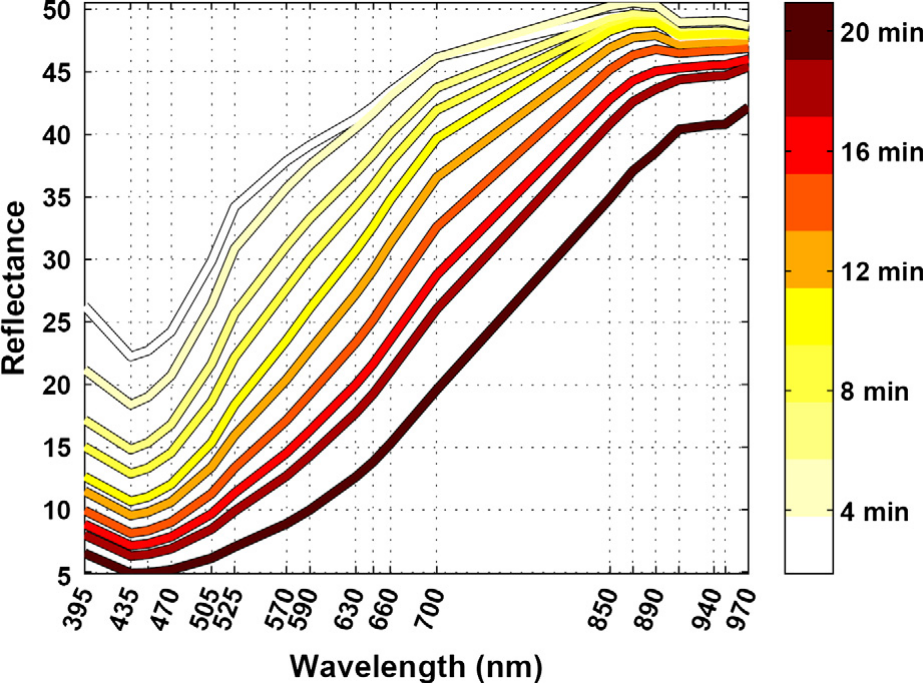
Spectra of training data. Each spectrum represents the mean spectral shape of all cookie pixels in a multispectral image. The shapes show a clear correlation with the baking time.

A clear correlation was seen between the baking time and mean spectra for the cookies (Fig. 2). The spectra are plotted as spectral reflectance values as a function of wavelength; they are colored according to the baking time. Light colors denote short baking times, dark colors, long baking times. The reflectance of the spectra decreases with baking time. This indicates that the lightness of the cookies is decreasing. The reflectance differences are largest between 400 and 700 nm (the visible wavelengths), while they are smaller in the NIR area. Decreasing steps in reflectance are seen in the short wave (400–500 nm) end of the spectrum. It is known that melanoids, the brown components formed from the Maillard reactions, have absorption properties in this area, specifically at 420 nm (Kim and Lee 2009).

### Browning evaluation and score

**Figure 3:**
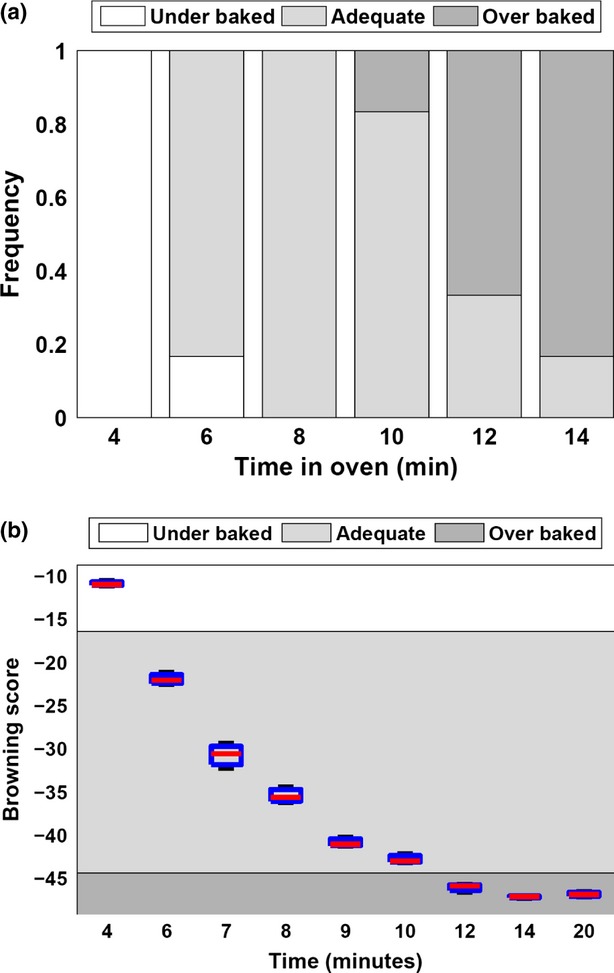
(a) Sensory evaluation. Each person classified a set of cookies into each of the stages underbaked, adequately baked, and overbaked. The histogram shows a normalized frequency for each group as a function of time in the oven. (b) Box-plot of mean values of browning score. Each box contains three samples. The background is shaded according to the classes defined by the sensory panel. The limits between the groups have been calculated as the mean euclidean distance between border boxes.

Results from the sensory evaluation of Set 1 are shown in Figure 3a. Cookies from each baking time were evaluated by each individual panelist; the evaluation shows that the adequate baking state is reached after 6 min.

**Figure 4:**
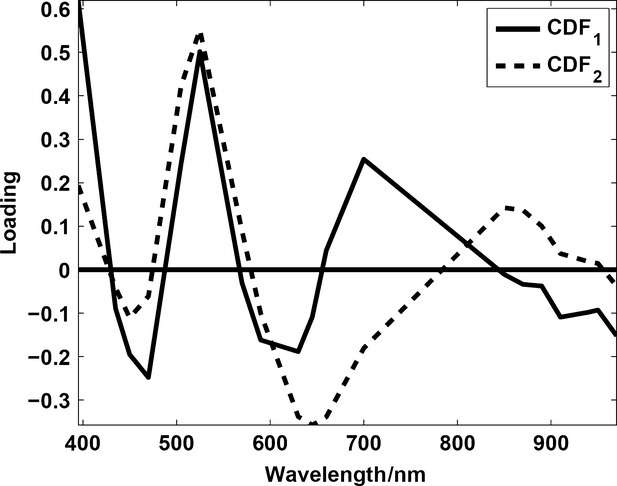
The loadings from the canonical discriminant analysis used to create the browning score. It is clearly seen how the area from 400 to 550 plays a significant role in the transformation vectors.

The panel generally agreed that baking times lower than 6 min resulted in underbaked cookies. Cookies baked from 6 to 10 min were considered adequately baked; cookies baked for more than 1 min were considered overbaked. No cookies were evaluated as belonging to all three classes. Neither were any cookies evaluated as belonging to both the under and overbaked categories. This indicates a sufficiently clear separation between the categories for the purpose of this study. The clear separation is also seen in Figure 3b quantifying the browning stages. In the figure a large gap between the extreme classes is shown as Euclidean distances based on the calculated browning scores. Figure 4 shows the two most significant projection vectors from the FDA.

**Figure 5:**
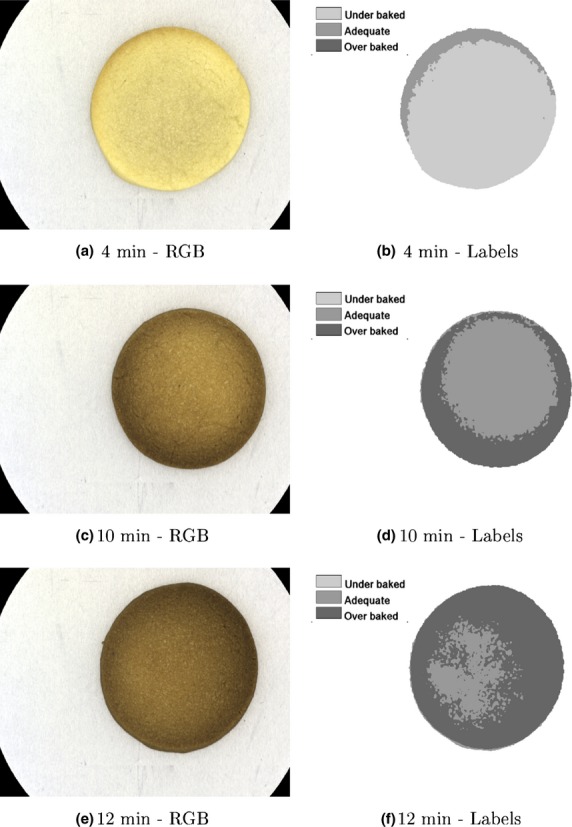
Pseudo-RGB representations of the multispectral images of cookies taken at different baking times. For each RGB image, a classified image is shown where pixels have been classified to one of the three browning stages. The predicted images were smoothed with a Gaussian filter before thresholding in order to remove noise. When observing the images from the top and down it is seen how the browning of the cookies starts from the edges and progress toward the center. Only the first cookie (4 min) contains areas of underbaked surface.

The most significant wavelengths in the first loading are 395 and 525 nm, both of which are seen to contribute largely to the variance in Figure 2. Each box in Figure 3b contains mean score values of the cookies used to train the FDA model together with the mean score values of the two other repetitions. The spread of the values is relatively low, while the development over time shows a significant decrease in the overall browning score. The limits between the classes were calculated as the mean between the browning scores for 4 and 6 min baking, and between 10 and 12 min. The browning score can be applied to multispectral recordings of new cookies to predict which stage of browning they are in. Examples are shown in Figure 5.

To check the uniformity of the browning on the surface of the cookies, pixel-wise browning scores were calculated. The result reveals how the browning propagates from the edge of the cookie toward the center during baking. Underbaked areas were only detected in cookies baked for 4 min; this corresponded well to the sensory evaluation. An overall browning score may be calculated for each cookie by taking the average of all the pixel-wise browning scores across the cookie.

**Figure 6:**
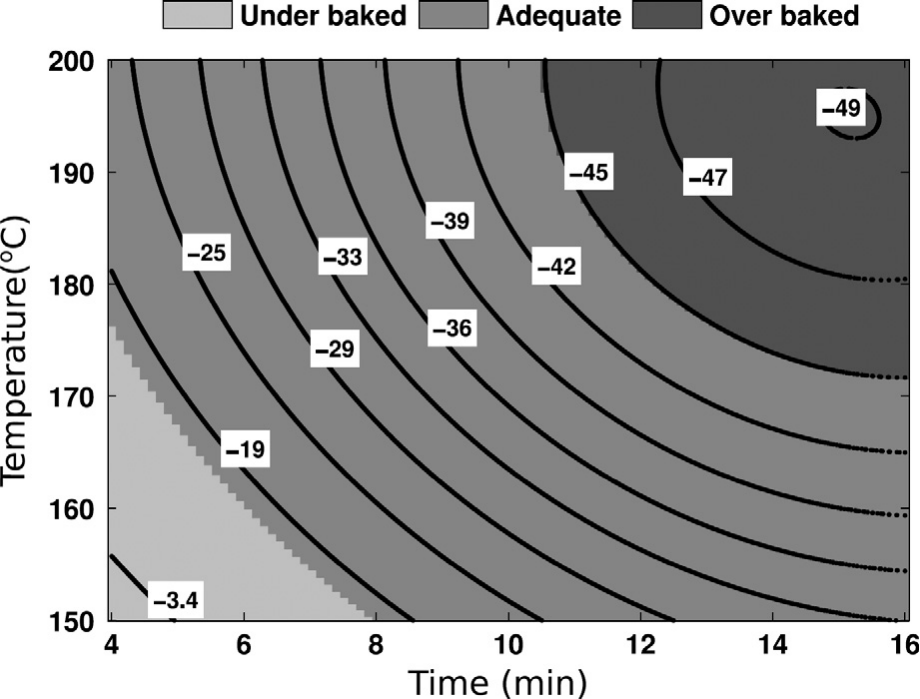
The surface browning score as a function of time and temperature for butter cookies. The zones indicate areas created by the sensory evaluation, corresponding to underbaked, adequately baked, and overbaked appearance.

The average browning scores were calculated for all cookies in Set 2 to assess the browning when varying both the baking time and the oven temperature. A quadratic surface was found to give the best description of the relation between browning and both oven temperature and baking time, without overfitting the data. Equation 3 was fitted to the browning scores, where *x*_1_ is the time (min) and *x*_2_ is the temperature (°C). The browning score value is 0 for no browning at the surface and decreases with increasing browning as shown in Figure 6.


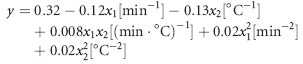
(3)

From the response surface, it is seen that a 1 min increase of the baking time will have approximately the same effect as increasing the baking temperature with 10°C. The results are valid within the investigated range of temperatures (150–200°C) and baking times (4–16 min), using the setup described in the Materials and Methods section. For other processes, new models should be developed, as the process equipment and setup influence the browning development strongly. The maximum baking time in the response surface was set to 16 min. Cookies were baked for longer baking times; up to 0 min, these are considered as extreme cases and are therefore not included in this part of the study.

### Determining water content

**Figure 7:**
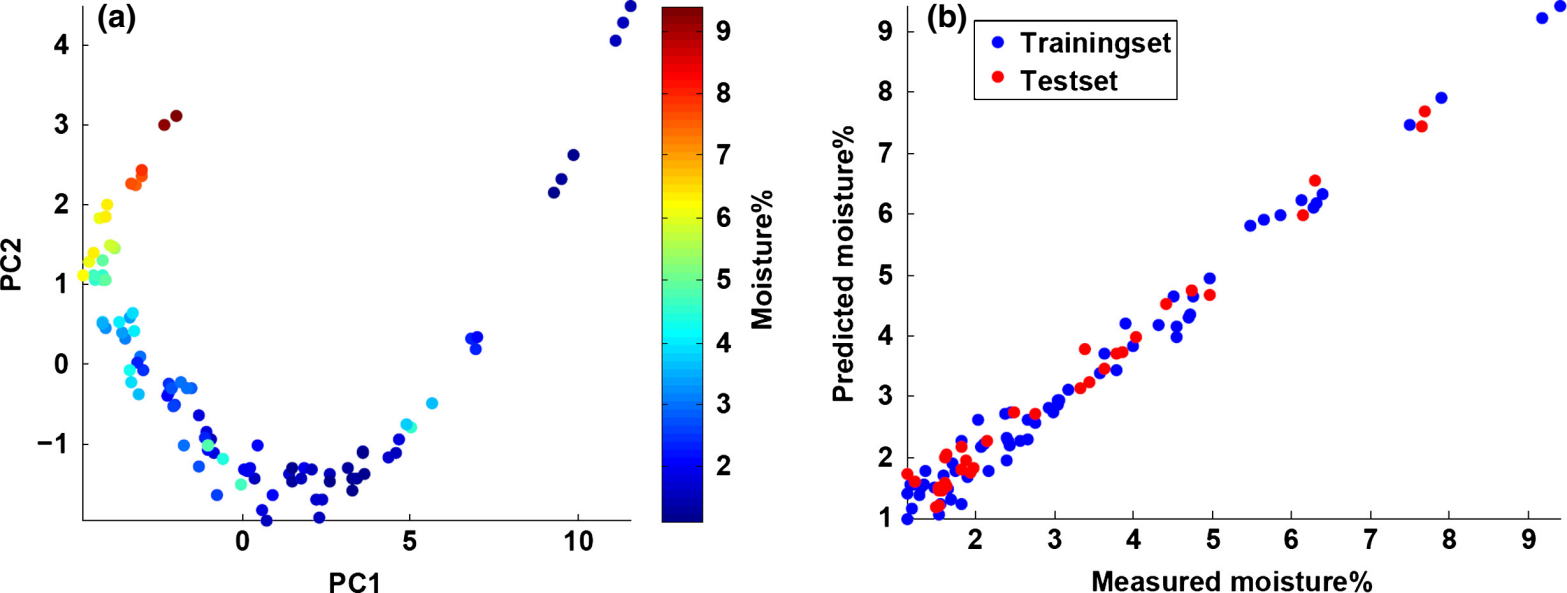
(a) Loadings of the water prediction model. The near-infrared area shows significant peaks together with the area around 470 nm. (b) Values of standard error of prediction calculated in a bootstrap procedure. The average of the squared error of prediction (SEP) values is calculated to 13.6, meaning that on average the prediction method has an error rate of 13.6%.

**Figure 8:**
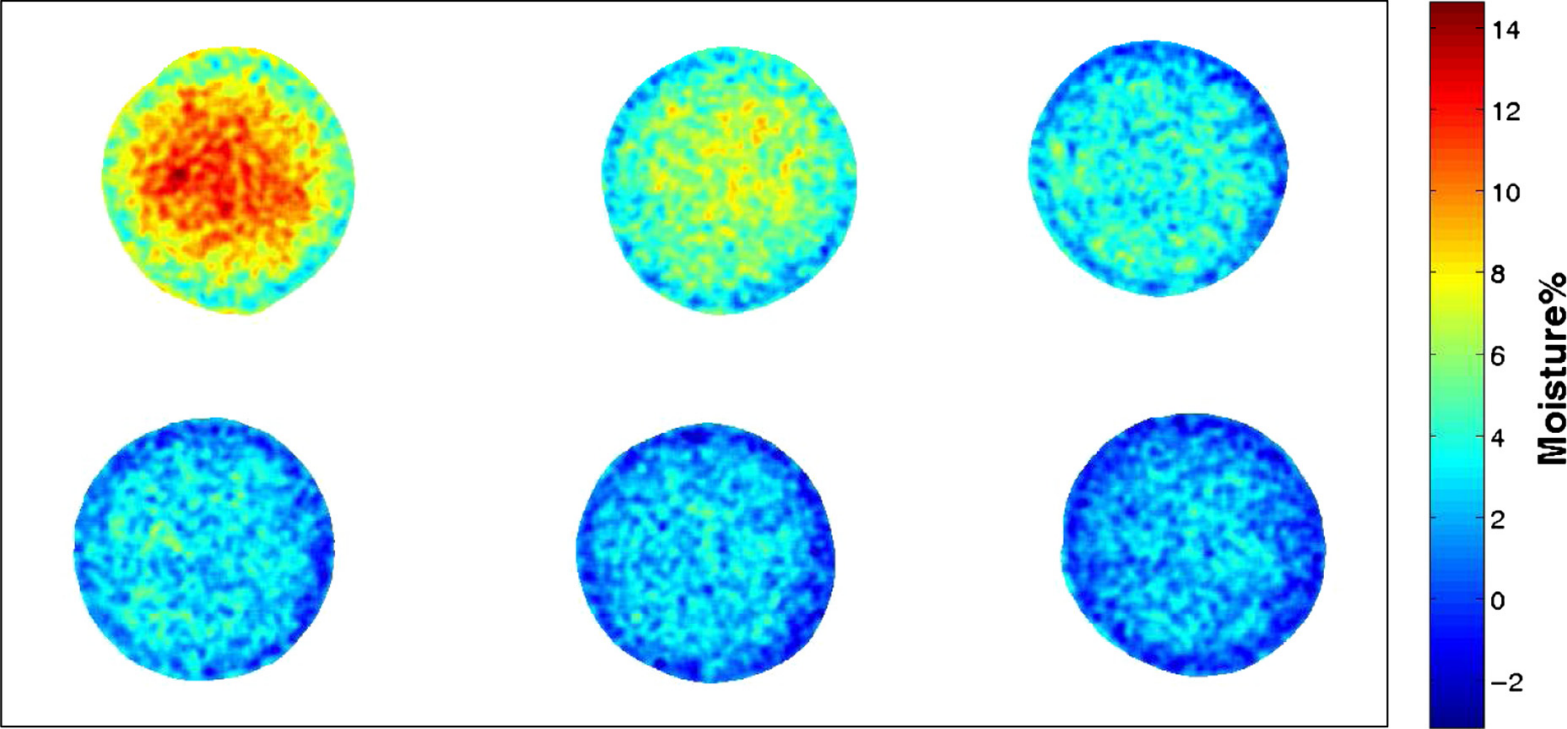
Water content for five cookies has been predicted for each pixel on the cookie surface and smoothed to remove noise. The average water prediction for the five cookies has been calculated and presented in Table 2. The color map used in the visualization of the five surfaces is the same where blue values correspond to low water content and red values correspond to high water content.

A total of 99 cookies were used for building and evaluating the model for average water content. Based on the 18 spectra obtained for each cookie surface, a mean value was calculated. A principal components analysis, which reveals the primary latent variance in the extracted features, is shown in Figure 7a. Each dot is a score value representing a cookie, color coded by its water content. Especially, PC 1 provides a good description of the development in the average water content. This variation was related to the measured water content using a partial least squares regression model. In order to build the model, 66 cookies were selected randomly and used in a fivefold cross-validation scheme for parameter estimation. Using the spectral means for training the model, PLSR components were calculated. From the model, the water content in the remaining 33 cookies was predicted. A plot of the predicted versus the measured values for both the training and the test cookies is seen in Figure 7b. This shows a very good approximation. Summing all the residuals, a RMSEP of 0.16% is obtained for the training set and 0.22% is obtained for the test set. The range of average water content in the data set spans from 1.13% to 9.39% (kg water per kg cookie). As expected, loadings from the model revealed that the short wave NIR area of the electromagnetic spectrum was highly correlated with the measured water content. The spatial distribution of water on the surface of each cookie was assessed by projecting each recorded spectrum of the cookie surface (one per pixel) with the calculated model. Figure 8 shows the spatial predictions on cookies baked at 150°C at different baking times. In Table 2, measurements and predicted values for the water content are shown for cookies baked at an oven temperature of 150°C and different baking times.

**Table 2:** Average real and predicted water content for the cookies the surfaces of which are shown in Figure 8. The real values have been measured using a drying cabinet while the predicted values were calculated as average values of all pixels on the surface

Time (min)	Temperature (°C)	Measured (%)	Predicted (%)	Deviation
4	150	9.17	9.18	0.01
6	150	6.40	6.30	−0.10
8	150	4.72	4.44	−0.28
10	150	3.57	3.60	0.03
12	150	2.38	2.75	0.37
14	150	2.07	2.24	0.17

## Conclusion

The used vision system records multispectral images in the visible and the first part of the NIR areas of the electromagnetic spectrum. An acceptance score was developed using FDA, based on the evaluation of cookies by a sensory panel. According to the sensory panel, cookies baked for 4 min were *underbaked*, while cookies baked for 6–10 min were *adequately baked*; longer baking times resulted in *overbaked* cookies. This evaluation was purely based on the visual appearance of the cookies, thus by the color and visual perception of texture.

The discriminant analysis revealed that the main wavelengths when creating the acceptance score were 395 and 525 nm, corresponding very well to the absorption properties of melanoids. From the acceptance score, a response surface was created, building a model which relates the influence of time and temperature to browning. The change in surface browning was comparable when changing the baking time with 1 min or the oven temperature with 10°C. A broad acceptance band was found, corresponding well to the sensory evaluations. A demonstration of the browning distribution on the surface of the cookies was presented showing how the browning starts from the edges of the cookie and works its way toward the center.

Besides assessing the surface browning, the water content in the cookies was evaluated. An approach similar to that of the acceptance score was used. The loading vector revealed that in order to map spectra to water content, the NIR area was of most importance. Having the NIR area in a water prediction model was expected due to the water peak around 970 nm. The water content was well estimated with a RMSEP of 0.22%.

It was shown that it is possible to use multispectral imaging to asses both the browning and the water content of butter cookies. Furthermore, the method was used to evaluate the browning and water content across the cookies surface, giving an indication of the evenness of the process and the produced products. In a production setting, only the relevant wavelengths would be used for monitoring, making image capturing and the following data processing possible during online production.

## Footnotes

1 Haas Meincke A/S, http://www.haas.com.

2 http://www.videometer.com, 12 June 2012.
